# Comment on 'Domestic light at night and breast cancer risk: a prospective analysis of 105 000 UK women in the Generations Study'

**DOI:** 10.1038/s41416-018-0060-7

**Published:** 2018-05-17

**Authors:** S. M. J. Mortazavi

**Affiliations:** 1 Department of Diagnostic Imaging, Fox Chase Cancer Center, 333 Cottman Ave, Philadelphia, PA 19111 USA; 2 Ionizing and Non-ionizing Radiation Protection Research Center (INIRPRC), Shiraz University of Medical Sciences, Shiraz, Iran

**Keywords:** Breast cancer, Risk factors, Epidemiology

This comment is regarding the article by Johns et al. entitled “Domestic light at night and breast cancer risk: a prospective analysis of 105 000 UK women in the Generations Study” published in the British Journal of Cancer^1^. Johns et al. have investigated the relationship between exposure to light at night (LAN) and breast cancer risk. Using questionnaires, these authors have collected data on the bedroom light levels and sleeping patterns of 105 866 participants who had no prior history of breast cancer. Their follow-up showed 1775 cases of breast cancer. In this prospective cohort analysis, Johns et al. concluded that there was no evidence of an association between LAN exposure and increased risk of breast cancer in their large UK-based cohort study. Despite its strengths, the paper authored by Johns et al. has at least two major shortcomings. Over the past decade, my colleagues and I have studied the adverse health effects of exposure to radiofrequency electromagnetic fields (RF-EMFs). Moreover, we have studied the biological effects of exposure to blue light emitted from digital screens^2, 3^. The first shortcoming of the paper authored by Johns et al. concerns the authors ignoring the key factor of melatonin suppression, caused by exposure to artificial short-wavelength blue light emitted from the very widely used digital screens (displays of smartphones, tablets, and laptops)^4^. It is worth noting that the study performed by the Pew research centre shows that about 95% of Americans own a cellphone^5^. It has recently been shown that blue light emitted from smartphones’ screens can be linked to decreased melatonin secretion (the mechanism behind the negative impact of smartphone use at night on sleep)^6^. We have previously shown that in people who use their smartphones at night, not only the RF-EMFs generated by smartphones but also the blue light emitted from the screens of these wireless devices can be associated with disrupted circadian rhythm^3^. Moreover, a recent study showed that the viewing distance of smartphones was negatively associated with subjective sleep status^6^. Therefore, it has been hypothesised that if women with hereditary breast cancer predispositions, such as women who carry mutated BRCA1 or BRCA2 or women with a family history of breast cancer, use their smartphones, tablets, and laptops at night, the disrupted circadian rhythms can amplify the risk of breast cancer^7^.

Another shortcoming of this study comes from its poor design. The authors asked women “*to report their level of exposure to LAN over the year prior to recruitment and at age 20 years, in the room in which they slept, in the categories; ‘light enough to read’; ‘light enough to see across the room, but not read’; ‘light enough to see your hand in front of you, but not to see across the room’; and ‘too dark to see your hand, or you wear a mask*’”. This study design shows that the authors have only focused on the intensity of light, while substantial data indicate that the spectral characteristics of light (wavelength) also significantly affect the melatonin level, sleep pattern, and circadian rhythms^8–13^. In summary, ignoring some key factors in the study conducted by Johns et al. has possibly affected the validity of their findings.

## References

[1] Johns LE (2018). Domestic light at night and breast cancer risk: a prospective analysis of 105 000 UK women in the Generations Study. Br. J. Cancer.

[2] Taheri M (2017). Exposure to visible light emitted from smartphones and tablets increases the proliferation of *Staphylococcus aureus*: can this be linked to acne?. J. Biomed. Phys. Eng..

[3] Mortazavi S, Mortazavi S, Habibzadeh P, Mortazavi G (2016). Is it blue light or increased electromagnetic fields which affects the circadian rhythm in people who use smartphones at night. Iran J. Public Health.

[4] Oh JH, Yoo H, Park HK, Do YR (2015). Analysis of circadian properties and healthy levels of blue light from smartphones at night. Sci. Rep..

[5] Pew Research Center. Mobile fact sheet (2017). http://www.pewinternet.org/fact-sheet/mobile/

[6] Yoshimura M (2017). Smartphone viewing distance and sleep: an experimental study utilizing motion capture technology. Nat. Sci. Sleep.

[7] Mortazavi SAR, Mortazavi SMJ (2018). Women with hereditary breast cancer predispositions should avoid using their smartphones, tablets and laptops at night. Iran. J. Basic Med. Sci..

[8] Bauer M (2018). The potential influence of LED lighting on mental illness. World J. Biol. Psychiatry..

[9] Shechter A, Kim EW, St-Onge MP, Westwood AJ (2018). Blocking nocturnal blue light for insomnia: A randomized controlled trial. J. Psychiatr. Res..

[10] Sletten TL (2017). Randomised controlled trial of the efficacy of a blue-enriched light intervention to improve alertness and performance in night shift workers. Occup. Environ. Med..

[11] Kozaki T, Kubokawa A, Taketomi R, Hatae K (2016). Light-induced melatonin suppression at night after exposure to different wavelength composition of morning light. Neurosci. Lett..

[12] Leung TW, Li RW, Kee CS (2017). Blue-light filtering spectacle lenses: optical and clinical performances. PLoS ONE.

[13] Ostrin LA, Abbott KS, Queener HM (2017). Attenuation of short wavelengths alters sleep and the ipRGC pupil response. Ophthalmic Physiol. Opt..

